# Development of an Electro-Responsive Sorafenib-Loaded Polypyrrole Coating-Modified Nickel–Titanium Alloy for Tumor Ablation Therapy

**DOI:** 10.3390/jfb17080405

**Published:** 2026-08-14

**Authors:** Lele Liu, Peng Gu, Dan Xia, Yonghao Wen, Baoe Li, Donghui Wang, Yaohong Wu

**Affiliations:** 1 Hebei Key Laboratory of Biomaterials and Smart Theranostics, School of Health Sciences and Biomedical Engineering, Hebei University of Technology, Tianjin 300130, China; y1939740522@163.com; 2 Department of Spine Surgery, Ganzhou People’s Hospital, Ganzhou 341006, China; gp2120746@163.com (P.G.); wenyonghao2021@163.com (Y.W.); 3 Tianjin Key Laboratory of Materials Laminating Fabrication and Interface Control Technology, School of Materials Science and Engineering, Hebei University of Technology, Tianjin 300130, China; xiad@hebut.edu.cn (D.X.); libaoe@hotmail.com (B.L.)

**Keywords:** nickel–titanium alloy, polypyrrole, ES, sorafenib, electrically controlled drug release, tumor ablation

## Abstract

Nickel–titanium (NiTi) stents have been widely used for the palliative management of portal vein tumor thrombosis (PVTT) due to their excellent mechanical strength and biocompatibility. However, conventional NiTi implants are therapeutically passive and lack intrinsic antitumor activity, rendering them vulnerable to tumor ingrowth and subsequent restenosis. Electrical stimulation-mediated ablation offers a controllable physical strategy for local tumor clearance; however, the native TiO_2_ passivation layer on the NiTi surface restricts its interfacial electroactivity. Electrochemical impedance spectroscopy revealed that the PPy coating significantly reduced the interfacial impedance of the NiTi substrate. After loading with sorafenib, NiTi−PPy−S maintained good electrochemical responsiveness and enabled voltage-dependent drug release characteristics. Under an applied potential of 0.9 V for 24 h, approximately 10.522 ± 0.295 μg/cm^2^ of the loaded sorafenib was released mechanistically, whereas only 0.249 ± 0.171 μg/cm^2^ was released through passive diffusion over the same period. The antitumor effect is closely associated with ES-triggered Ca^2+^ influx, mitochondrial Ca^2+^ overload, loss of mitochondrial membrane potential, and caspase-3-dependent apoptosis. These findings demonstrate that electro-responsive PPy can transform passive NiTi implants into active antitumor therapeutic interfaces, offering a promising strategy for developing multifunctional implants for PVTT treatment.

## 1. Introduction

Portal vein tumor thrombosis (PVTT) is a frequent and life-threatening complication of advanced hepatocellular carcinoma (HCC) [1,2]. Owing to the high propensity of HCC for vascular invasion, tumor cells may infiltrate the portal venous system, resulting in portal vein obstruction, impaired hepatic blood flow, and portal hypertension. For patients who are not eligible for curative surgical resection, portal vein stent implantation provides an important palliative approach to restoring portal venous flow, relieving portal hypertension, and maintaining vascular patency.

Nickel–titanium (NiTi) alloy stents, characterized by excellent biocompatibility and mechanical strength, have been widely applied in clinical practice; notably, previous studies have demonstrated that NiTi-based portal vein stenting combined with iodine-125 seed strand implantation and transcatheter arterial chemoembolization is a feasible therapeutic strategy for hepatocellular carcinoma patients with portal vein tumor thrombus (PVTT) [3]. The use of these stents to dilate occluded sites has become an emerging palliative strategy for treating PVTT [4]. Electroablation (ES), which induces irreversible tumor cell damage through the application of external electrical pulses delivered by implanted electrodes, represents a physical therapeutic strategy capable of triggering immunogenic cell death, leading to the release of damage-associated molecular patterns (DAMPs) and subsequent activation of antitumor immune responses [5,6]. Because NiTi alloy stents are inherently conductive, they hold promise for use as tumor ablation electrodes during interventional procedures while simultaneously dilating the occlusion.

However, due to the inherent lack of anticancer activity in NiTi alloys [7], residual tumor tissue around the implant is difficult to completely remove. This limitation makes the stents highly susceptible to re-occlusion caused by tumor re-invasion [8], thereby severely limiting their long-term efficacy. Therefore, there is an urgent need to develop a novel stent treatment modality that can efficiently eliminate tumor cells while ensuring safety.

Electroactive surface modification represents an effective approach to addressing the limitations described above. Polypyrrole (PPy) is one of the most extensively investigated conductive polymers and has been widely explored for biomedical bioelectrodes due to its electrical conductivity, electrochemical activity, and favorable biological compatibility [9], biosensors [10], and electrically triggered drug-delivery systems [11], owing to its high electrical conductivity [12], chemical stability [13], biocompatibility [14], and reversible doping/dedoping behavior [15].

The conductive polymer polypyrrole system can be used for the on-demand release of anionic [16], cationic [17,18], and neutral drugs [19]. The surfactant sodium dodecylbenzenesulfonate (SDBS) enhances the dispersion and effective encapsulation of sorafenib within the conductive polymer matrix. This strategy thereby establishes an important link between pharmaceutical formulation principles and electrochemical materials fabrication, facilitating the development of drug-functionalized electroactive coatings. During the oxidative electropolymerization of pyrrole, anionic dopants are incorporated into the growing PPy coating to compensate for the positive charges generated along the polymer backbone. In the present system, DBS^−^ derived from SDBS serves as the principal charge-compensating dopant. Sorafenib is not incorporated as an ionic dopant; instead, Sorafenib is primarily physically encapsulated within surfactant micelles formed by SDBS, a process that may be further facilitated by hydrophobic interactions and other noncovalent interactions within the polymer–surfactant matrix. Upon ES, redox-induced changes in the PPy coating, accompanied by ion transport and changes in the polymer structure, may facilitate the release of the physically incorporated sorafenib [20]. These redox-driven transport processes may induce reversible swelling or contraction of the polymer matrix, rearrangement of the PPy chains, and changes in the hydration state, free volume, and local porosity. Specifically, although conductive polymer coatings have been extensively investigated for biomedical applications, the efficient incorporation of poorly water-soluble hydrophobic therapeutic agents into electrochemically deposited polymer matrices remains challenging.

Building on these considerations, sorafenib-loaded polypyrrole coatings, denoted NiTi−PPy−S, were fabricated on NiTi alloy surfaces by electrochemical deposition. First, the electrodeposition parameters were optimized to fabricate PPy coatings with favorable electrochemical activity and structural stability. Subsequently, multiple physicochemical characterization methods confirmed that the sorafenib was successfully incorporated into the conductive PPy network. The electroresponsive coating was then evaluated for its ability to enhance interfacial charge transfer, mediate voltage-dependent sorafenib release, and achieve synergistic antitumor activity.

## 2. Materials and Methods

### 2.1. Chemical Reagents

All chemicals and reagents were of analytical grade, including pyrrole (Py, Aladdin, Shanghai, China), sodium dodecylbenzenesulfonate (SDBS, Aladdin, Shanghai, China), acetone, ethanol, hydrofluoric acid (HF, Jiangtian Chemical Technology Co., Ltd., Tianjin, China), nitric acid (HNO_3_, Jiangtian Chemical Technology Co., Ltd., Tianjin, China), and phosphate-buffered saline (PBS, Beyotime, Shanghai, China). The pyrrole monomer was purified by vacuum distillation and subsequently stored at 4 °C in the dark prior to use. Murine hepatoma cells (Hepa1-6, Procell, Wuhan, China) were used to evaluate antitumor activity, whereas human umbilical vein endothelial cells (HUVECs, Procell, Wuhan, China) were used to assess endothelial cytocompatibility.

#### 2.1.1. Pretreatment of NiTi Substrates

NiTi sheets (Commercial, Shanghai, China) were machined into circular discs measuring 20 mm in diameter and 2 mm in thickness. The discs were sequentially ground with 600-, 800-, 1200-, and 2000-grit silicon carbide abrasive papers to remove surface contaminants, irregularities, and the pre-existing oxide layer. The samples were then ultrasonically cleaned sequentially in acetone, ethanol, and deionized water. To further clean and activate the surface, the NiTi discs were etched for 5 min in an HF/HNO_3_/deionized water solution at a volume ratio of 1:5:34, followed by ultrasonic cleaning in ethanol and deionized water. Finally, the pretreated samples were dried at room temperature and stored until further use.

#### 2.1.2. Preparation of NiTi-PPy Coatings

PPy coatings were electrodeposited on the pretreated NiTi substrates using an electrochemical workstation (Chenhua Instruments Co., Ltd., Shanghai, China) in a conventional three-electrode configuration. A pretreated NiTi disc, a saturated calomel electrode (SCE, Shanghai Huayu Instruments & Meters, Shanghai, China), and a platinum foil (Shanghai Huayu Instruments & Meters, Shanghai, China) were used as the working, reference, and counter electrodes, respectively. Purified Py was added to an SDBS solution to achieve final concentrations of 0.1 M Py and 0.04 M SDBS, and the resulting solution was stirred for 1 h in the dark to prepare the electropolymerization electrolyte. Based on preliminary optimization, potentiostatic electrodeposition was performed at 0.7 V versus SCE for 500 s. Following deposition, the samples were thoroughly rinsed with deionized water to remove residual monomers, soluble oligomers, and loosely bound species, and subsequently dried at 37 °C. The resulting samples were designated NiTi−PPy.

#### 2.1.3. Preparation of Sorafenib-Loaded NiTi-PPy Coatings

To prepare NiTi−PPy−S, an aqueous SDBS solution (0.04 M) was first prepared. Sorafenib was subsequently added to the SDBS solution and ultrasonicated for 30 min, followed by magnetic stirring for 1 h, resulting in a homogeneous, turbid suspension for subsequent electrodeposition. PPy electrodeposition was then performed using the same three-electrode configuration and deposition conditions described above. Following deposition, the samples were thoroughly rinsed several times with deionized water to remove residual monomers and loosely adsorbed sorafenib. The resulting samples were designated NiTi−PPy−S. The sample abbreviations and corresponding experimental groups are summarized in Table 1.

### 2.2. Characterization of Material Surface Structure and Composition

#### Morphological Characterization, Chemical Structure, and Compositional Analysis

The surface morphologies of NiTi, NiTi−PPy, and NiTi−PPy−S samples were examined using scanning electron microscopy (SEM). Before imaging, the samples were mounted on SEM specimen stubs and sputter-coated with a thin layer of gold to improve surface conductivity and minimize charging effects. SEM images were acquired at an accelerating voltage of 15 kV.

Fourier transform infrared spectroscopy (FTIR) was employed to identify the characteristic functional groups and analyze the chemical structures of the samples over the wavenumber range of 4000–400 cm^−1^. X-ray diffraction (XRD) was used to characterize the phase composition and crystalline structure of the NiTi substrate and the deposited coatings. X-ray photoelectron spectroscopy (XPS) was performed to determine the surface elemental composition and chemical states of the constituent elements, with the binding energies calibrated against the C 1s peak. Zeta potential measurements were conducted to evaluate changes in sample surface charge following sorafenib loading.

### 2.3. Electrochemical Properties and Conductivity Testing

Electrochemical measurements were performed at room temperature in 0.01 M phosphate-buffered saline (PBS) using a conventional three-electrode configuration. Before each measurement, the samples were immersed in PBS for 1 h to allow the open-circuit potential (OCP) to reach a stable value. Cyclic voltammetry (CV) was conducted to evaluate the electrochemical redox behavior of the coatings. Electrochemical impedance spectroscopy (EIS) was performed to assess interfacial charge-transfer characteristics over a frequency range of 100 kHz to 0.1 Hz, using a sinusoidal AC perturbation with an amplitude of 10 mV.

### 2.4. Electro-Responsive Release of Sorafenib

A calibration curve for sorafenib was first established by measuring the absorbance of standard sorafenib solutions at the characteristic maximum absorption wavelength. For the electrically triggered drug-release assay, NiTi−PPy−S samples were immersed in 0.9% (*w*/*v*) NaCl solution and subjected to applied potentials of 0, 0.3, 0.6, or 0.9 V. At predetermined time points, aliquots of the release medium were collected, and the concentration of released sorafenib was quantified by ultraviolet–visible (UV–Vis) spectrophotometry using the established calibration curve.

### 2.5. ES Parameters

ES was delivered using a DC power supply operating in constant-voltage mode at 0.7 V for 20 min. During stimulation, the current gradually increased, reaching approximately 500 mA at the end of the stimulation period.

### 2.6. Biological Evaluation of Materials

#### 2.6.1. Cell Culture

Murine hepatoma cells (Hepa 1-6, Procell, Wuhan, China) (Catalog No.: CL-0105; deposited at ATCC: CRL-1830, BCRC: 60051, DSMZ: ACC-175, ECACC: 92110305), and human umbilical vein endothelial cells (HUVECs, Procell, Wuhan, China) were purchased from Wuhan Punose Life Technology Co., Ltd. (Catalog No.: CP-H082).

Hepa1-6 cells and HUVECs were cultured in the appropriate complete culture media at 37 °C in a humidified incubator containing 5% CO_2_. The culture media were replaced as required according to cell confluence, and the cells were subcultured every 2–3 days.

#### 2.6.2. Cytotoxicity and Proliferation Assays

Hepa1-6 cells and HUVECs were separately seeded onto the surfaces of NiTi, NiTi-PPy, and NiTi-PPy-S samples. To evaluate the biocompatibility of the materials, the cells were cultured on the different sample surfaces in the absence of ES. To assess the cellular effects of ES, cells cultured on each sample surface were subsequently subjected to ES. Cell viability and membrane integrity were assessed using calcein acetoxymethyl ester/propidium iodide (Calcein-AM/PI) live/dead staining. Cellular metabolic activity and relative viability were further quantified at predetermined time points using the Cell Counting Kit-8 (CCK-8) assay.

#### 2.6.3. Apoptosis Analysis

Apoptosis was quantified by Annexin V-fluorescein isothiocyanate/propidium iodide (Annexin V-FITC/PI) dual staining followed by flow cytometric analysis. Hepa1-6 cells and HUVECs were separately cultured on the surfaces of the different samples. Following treatment in the presence or absence of ES, the cells were harvested, washed with PBS, and stained in accordance with the manufacturer’s instructions. The percentages of viable cells, early apoptotic cells, late apoptotic or secondary necrotic cells, and necrotic cells were determined based on Annexin V-FITC and PI fluorescence.

#### 2.6.4. Mitochondrial Membrane Potential

The mitochondrial membrane potential (Δψ) of Hepa1-6 cells and HUVECs after ES was assessed using JC-1 fluorescent staining. In cells with intact mitochondrial membrane potential, JC-1 accumulates within the mitochondrial matrix and forms aggregates that emit red fluorescence. In contrast, mitochondrial depolarization prevents JC-1 aggregation, resulting in the predominance of monomers that emit green fluorescence. Accordingly, a decrease in the red-to-green fluorescence intensity ratio was considered indicative of mitochondrial membrane depolarization.

#### 2.6.5. Intracellular Ca^2+^ Detection

Intracellular Ca^2+^ levels were evaluated using the fluorescent calcium indicator Fluo-4 acetoxymethyl ester (Fluo-4 AM). Cells were cultured on the surfaces of the different samples and subjected to ES. The cells were subsequently incubated with Fluo-4 AM working solution, and intracellular Ca^2+^-associated fluorescence was visualized by confocal laser scanning microscopy.

#### 2.6.6. Mitochondrial Ca^2+^ Assay

Mitochondrial Ca^2+^ accumulation was evaluated using the mitochondria-targeting fluorescent calcium indicator Rhod-2 acetoxymethyl ester (Rhod-2 AM). Following ES, the cells were incubated with Rhod-2 AM working solution, and mitochondrial Ca^2+^-associated fluorescence was visualized by fluorescence microscopy. Changes in fluorescence intensity were used to assess relative mitochondrial Ca^2+^ accumulation among the experimental groups.

#### 2.6.7. RT-qPCR Analysis

Following ES, total RNA was isolated from cells cultured on the surfaces of the different samples. The extracted RNA was reverse-transcribed into complementary DNA (cDNA), and the mRNA expression levels of apoptosis-related genes, including Caspase-3 and Bcl-2, were determined by quantitative reverse-transcription polymerase chain reaction (RT-qPCR). Relative gene expression was normalized to a reference gene and calculated using the 2^−ΔΔCt^ method.

### 2.7. In Vivo Tumor Ablation

A subcutaneous Hepa1-6 tumor model was established in BALB/c nude mice. After successful tumor establishment, the mice were randomly assigned to six groups: NiTi without ES (−ES−NiTi), NiTi−PPy without ES (−ES−NiTi−PPy), NiTi−PPy−S without ES (−ES−NiTi−PPy−S), NiTi with ES (+ES−NiTi), NiTi-PPy with ES (+ES−NiTi−PPy), and NiTi−PPy−S with ES (+ES−NiTi−PPy−S). A sterilized circular implant measuring 20 mm in diameter and 2 mm in thickness, corresponding to the assigned treatment, was surgically placed beneath each tumor and maintained in direct contact with the tumor base to facilitate localized drug delivery and ES. Mice in the +ES groups were subjected to ES at 0.9 V for 20 min once daily, whereas those in the −ES groups underwent the same implantation procedure but received no ES. Tumor volume and body weight were monitored throughout the treatment period, and tumor weight was determined after excision at the experimental endpoint. On day 13, all mice were euthanized, and the tumors and major organs were collected for subsequent histopathological examination.

### 2.8. Histology and Immunofluorescence Staining

Tumor tissues were fixed in 4% paraformaldehyde, paraffin-embedded, and cut into sections. Hematoxylin and eosin (H&E) staining was performed to evaluate histopathological changes, including tumor architecture and necrosis, whereas Masson’s trichrome staining was used to assess tissue organization and collagen deposition. Terminal deoxynucleotidyl transferase dUTP nick-end labeling (TUNEL) staining was performed to identify cells exhibiting apoptosis-associated DNA fragmentation within the tumor tissues. Major organs, including the heart, liver, spleen, lungs, and kidneys, were also subjected to H&E staining to evaluate potential systemic toxicity and histopathological abnormalities.

### 2.9. Ethics Approval

All animal experimentation protocols were approved by the Ethics Review Committee of Hebei University of Technology (Approval No.: HEBUTacvc2023040) and were conducted in accordance with the “Guidelines for the Humane Treatment of Laboratory Animals” issued by the Ministry of Science and Technology of China, as well as relevant international ethical principles for animal experimentation.

### 2.10. Statistical Analysis

All quantitative data were presented as the mean ± standard deviation (SD). Comparisons among multiple groups were performed using one-way analysis of variance (ANOVA), followed by an appropriate post hoc multiple-comparison test. Statistical significance was set at * *p* < 0.05, with significance levels denoted as follows: * *p* < 0.05, ** *p* < 0.01, *** *p* < 0.001, and **** *p* < 0.0001.

## 3. Results and Discussion

### 3.1. Morphology, Structure, and Electrochemical Properties of the Coating

**Figure 1:**
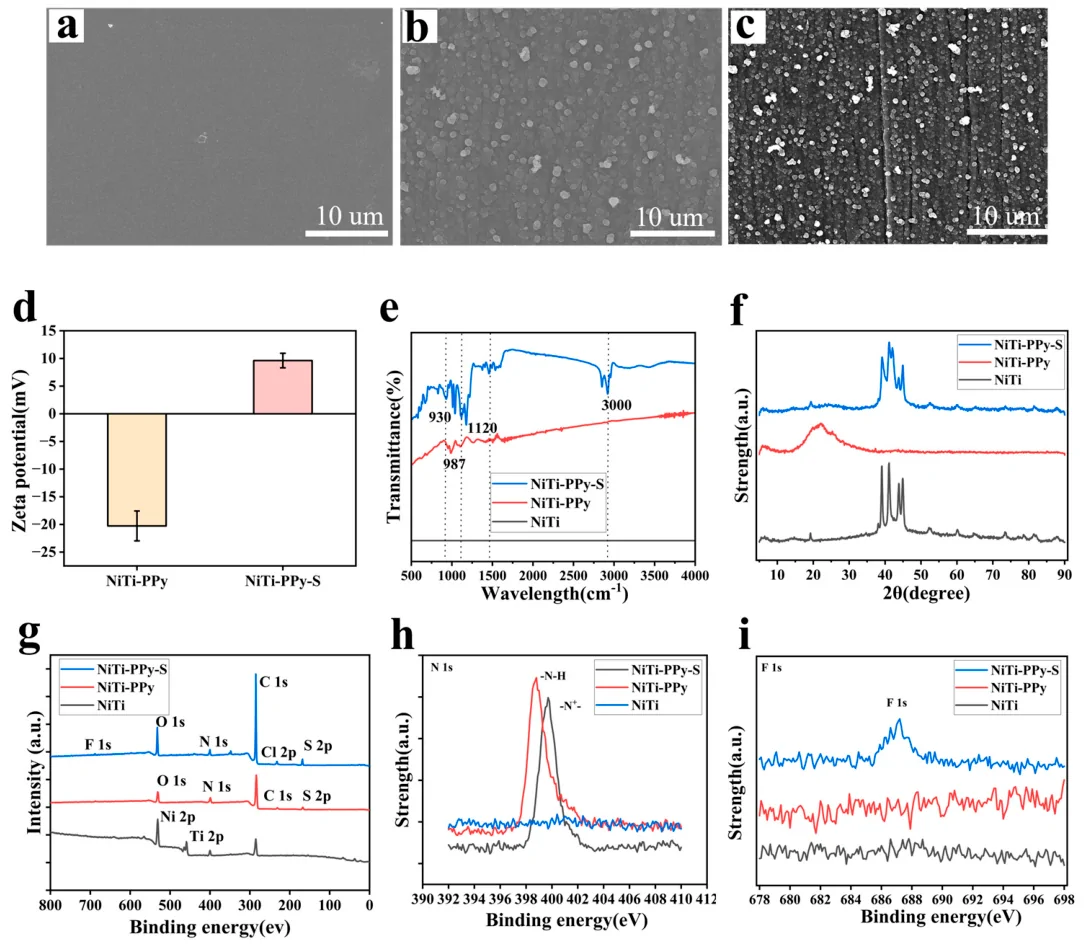
(**a**) SEM image of NiTi; (**b**) SEM image of NiTi−PPy; (**c**) SEM image of NiTi−PPy−S sample; (**d**) Zeta potential plots of NiTi−PPy and NiTi−PPy−S samples; (**e**) FT-IR spectra of NiTi, NiTi−PPy, and NiTi−PPy−S samples; (**f**) XRD patterns of NiTi, NiTi−PPy, and NiTi−PPy−S samples; (**g**) XPS patterns of NiTi, NiTi−PPy, and NiTi−PPy−S samples; (**h**) high-resolution N 1s spectra; (**i**) high-resolution F 1s spectra of NiTi, NiTi−PPy, and NiTi−PPy−S samples.

Figure 1 shows the surface morphologies and physicochemical characteristics of the NiTi, NiTi−PPy, and NiTi−PPy−S samples. As shown in Figure 1a, the polished NiTi substrate exhibited a relatively smooth surface. Figure 1b shows that, following PPy electrodeposition, the NiTi surface was uniformly covered by a PPy coating exhibiting a characteristic cauliflower-like microstructure. When sorafenib is incorporated into the PPy coating network, marked changes in the surface morphology were observed. As shown in Figure 1c, numerous fine, bright particulates were distributed over the rounded cauliflower-like PPy structures, which suggested that the presence of sorafenib affected the electropolymerization and deposition behavior of PPy and was consistent with its incorporation into the coatings.

Zeta potential measurements further revealed changes in the surface charge characteristics following sorafenib loading. As shown in Figure 1d, NiTi−PPy exhibited a negative zeta potential, whereas NiTi−PPy−S displayed a shift toward positive values. This charge reversal suggested an alteration in the surface chemical environment arising from interactions among sorafenib, PPy, and the dopant species. The change may be associated with the ionization state and surface orientation of sorafenib, as well as the masking or redistribution of negatively charged sites within the PPy coating. Collectively, the morphological and zeta potential results supported the successful incorporation of sorafenib into the PPy-based coating.

Figure 1e presents the FTIR spectra of the different samples, providing further evidence for PPy deposition and sorafenib incorporation. The absorption bands in the range of 1540–1450 cm^−1^ were assigned to the characteristic C=C and C–N stretching vibrations of the pyrrole ring, whereas the band at approximately 987 cm^−1^ was attributed to the out-of-plane C–H deformation vibration of PPy [21]. These characteristic bands supported the successful electropolymerization of PPy on the NiTi substrate. The bands located at approximately 1100–1200 cm^−1^ may arise from vibrational modes of the benzene sulfonate groups in SDBS, together with in-plane deformation vibrations of PPy. Following sorafenib loading, the spectrum of NiTi−PPy−S exhibited additional absorption features attributable to sorafenib. The bands in the region near 3000 cm^−1^ were associated with C–H stretching vibrations of aromatic and aliphatic groups. The absorption features near 1120 cm^−1^ may be related to C–F vibrations of the trifluoromethyl group in sorafenib, whereas the band at approximately 930 cm^−1^ may originate from aromatic ring deformation or other sorafenib-associated vibrational modes [22]. Collectively, the changes in the FTIR spectra were consistent with the incorporation of sorafenib into the PPy coating.

Figure 1f presents the XRD patterns of the different samples. The NiTi substrate exhibited prominent diffraction peaks in the 2θ range of approximately 40–45°, which were consistent with the characteristic diffraction features of NiTi reported in previous studies [23]. These well-defined peaks indicated the crystalline nature of the NiTi substrate. In contrast, NiTi−PPy exhibited a broad diffraction halo centered at approximately 22–25°, accompanied by a marked reduction in the intensity of the characteristic NiTi reflections. The broad halo was attributed to the predominantly amorphous structure of PPy and the short-range ordering of polymer chains [24], including interactions among adjacent pyrrole units. The attenuation of the NiTi diffraction peaks suggested substantial surface coverage by the PPy coating, although the extent of peak attenuation may also have been influenced by coating thickness and the XRD measurement configuration [25]. After sorafenib incorporation, the NiTi−PPy−S sample displayed a more complex diffraction profile, which may be associated with changes in molecular packing, short-range ordering, or structural heterogeneity caused by drug incorporation [26]. These changes may reflect alterations in the molecular packing, short-range ordering, or structural heterogeneity of the PPy coating induced by sorafenib incorporation. In conjunction with the SEM observations, the XRD results suggested that sorafenib modified the organization of the PPy matrix and contributed to the formation of a structurally heterogeneous composite coating.

XPS provided further evidence for the formation of the PPy coating and the incorporation of sorafenib. As shown in Figure 1g, the appearance of a distinct N 1s signal after PPy electrodeposition indicated the presence of the nitrogen-containing PPy structure on the NiTi surface [21]. Following sorafenib loading, changes in the relative proportions of the different nitrogen species were observed, suggesting that sorafenib incorporation altered the local chemical environment and doping state of the PPy chains. The high-resolution N 1s spectrum of NiTi−PPy−S exhibited a broader and more complex profile than that of NiTi−PPy (Figure 1h). The N 1s envelope could be deconvoluted into components assigned to neutral pyrrolic nitrogen, positively charged or protonated nitrogen species, and other nitrogen-containing chemical environments associated with PPy and sorafenib. In particular, the increased contribution in the region of approximately 401–402 eV may reflect changes in positively charged nitrogen species or interfacial interactions between sorafenib and the PPy network [27]. These changes may arise from electrostatic interactions, hydrogen bonding, and sorafenib-induced modulation of the PPy doping state. In addition, nitrogen-containing groups in sorafenib, including urea, amide, and heterocyclic nitrogen species, may overlap with the N 1s signals of PPy, thereby contributing to the increased spectral complexity. As shown in Figure 1i, NiTi−PPy−S exhibited a distinct F 1s peak at approximately 687–688 eV, whereas no corresponding fluorine signal was detected for NiTi−PPy. Because fluorine is present in the trifluoromethyl group of sorafenib but absent from the PPy coating, the appearance of the F 1s signal provided strong evidence for the successful incorporation of sorafenib into the PPy-based coating. The high-resolution F 1s spectrum of the sorafenib-containing PPy coating exhibits a distinct signal at approximately 687 eV, which can be attributed to organic fluorine species, particularly the trifluoromethyl group of sorafenib [28]. Because pristine PPy does not contain fluorine, the presence of this F 1s signal provides evidence for the successful incorporation of sorafenib into the PPy coating. Separately, the high-resolution N 1s spectrum reflects the chemical states of nitrogen within the PPy backbone. Compared with pristine PPy, the N 1s peak of the sorafenib-containing coating is broadened and can be deconvoluted into components assigned to neutral pyrrolic nitrogen and other nitrogen species associated with the doped or oxidized states of PPy.

### 3.2. Electrochemical Properties

**Figure 2:**
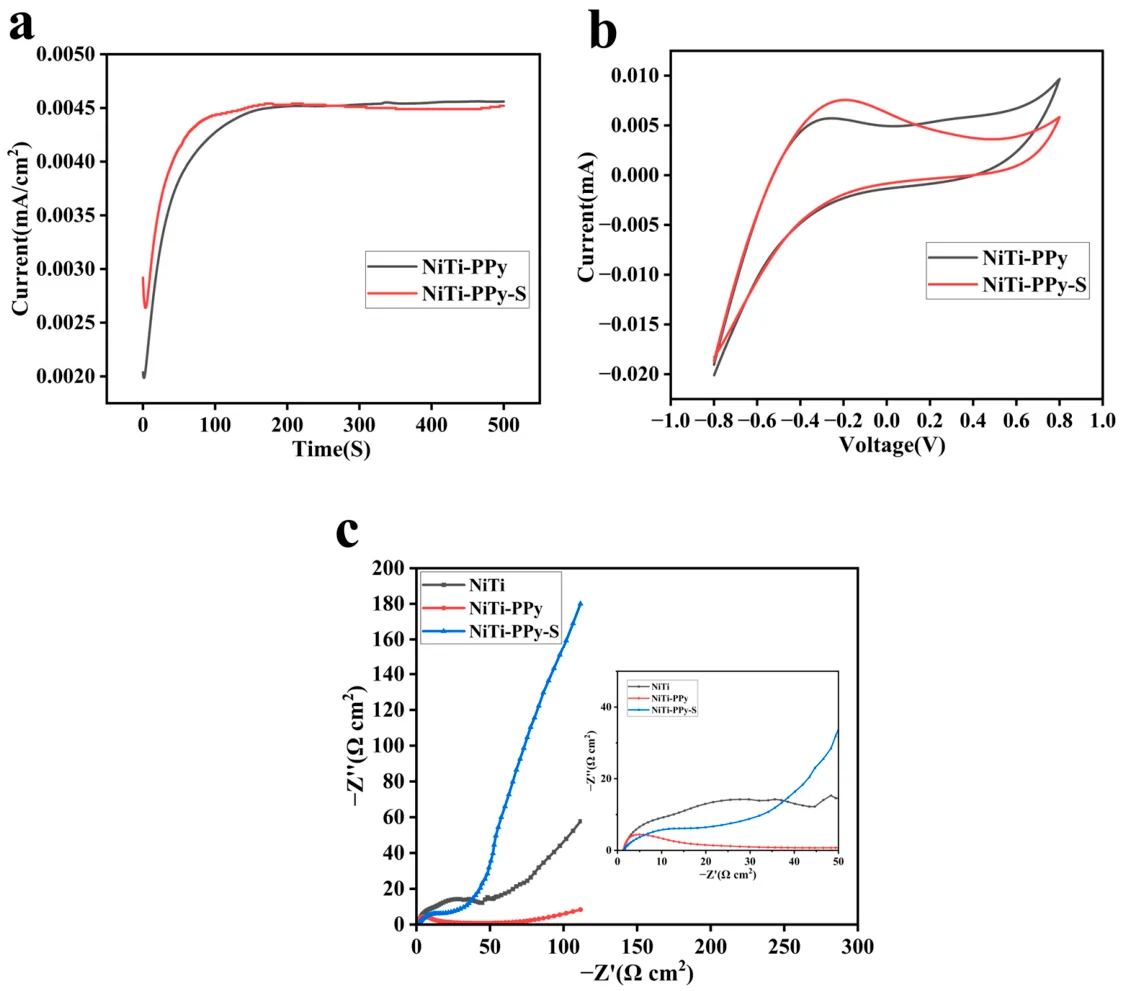
(**a**) Current–time (I-t) curves of NiTi−PPy and NiTi−PPy−S; (**b**) Redox Cyclic Voltammetry Curves of NiTi−PPy and NiTi−PPy−S samples; (**c**) Nyquist plots of NiTi, NiTi−PPy, and NiTi−PPy−S.

The current–time profiles recorded during the electrodeposition of NiTi−PPy and NiTi−PPy−S are shown in Figure 2a. The initial decrease in current was primarily associated with double-layer charging and transient interfacial polarization at the electrode surface. The subsequent rapid increase in current indicated the oxidation of Py monomers and the nucleation and growth of PPy on the NiTi substrate [29]. As polymerization proceeds, the PPy nuclei gradually expand and interconnect to form a continuous coating [30]. The current eventually stabilizes, indicating that the coating has a quasi-steady growth stage. Compared to NiTi−PPy, NiTi−PPy−S exhibits a more rapid increase in current during the early deposition stage. This difference may be associated with sorafenib-induced changes in the ionic environment of the electrolyte or interfacial adsorption behavior, which could affect PPy nucleation and growth kinetics [31]. The electrochemical performance of NiTi−PPy−S was further evaluated using CV and EIS. The CV curve in Figure 2b shows that NiTi-PPy-S still exhibits significant redox activity, indicating that the loading of sorafenib did not impair the electrochemical responsiveness of PPy. A more pronounced oxidation peak appears near approximately −0.42 V, which may be related to changes in coating morphology and internal free volume following drug incorporation, thereby facilitating ion transport within the PPy matrix [18]. The EIS results in Figure 2c reveal that bare NiTi exhibits the largest semicircle, which can be attributed to the formation of a TiO_2_-dominated passive layer and the resultant high interfacial charge-transfer resistance [32,33]. The NiTi−PPy sample exhibited the smallest semicircular arc, indicating that the conductive PPy coating significantly reduced interfacial impedance and improved ion/electron transport. The semicircular arc of the NiTi−PPy−S sample was larger than that of NiTi−PPy but still superior to that of bare NiTi, suggesting that the non-conductive sorafenib molecules partially hindered electron and ion transport without eliminating the coating’s electroactive function. Collectively, these findings demonstrated that NiTi−PPy−S retained sufficient electroactivity to function as an active interface for ES-mediated treatment and electrically triggered drug release.

### 3.3. Electrically Controlled Sorafenib Release

**Figure 3:**
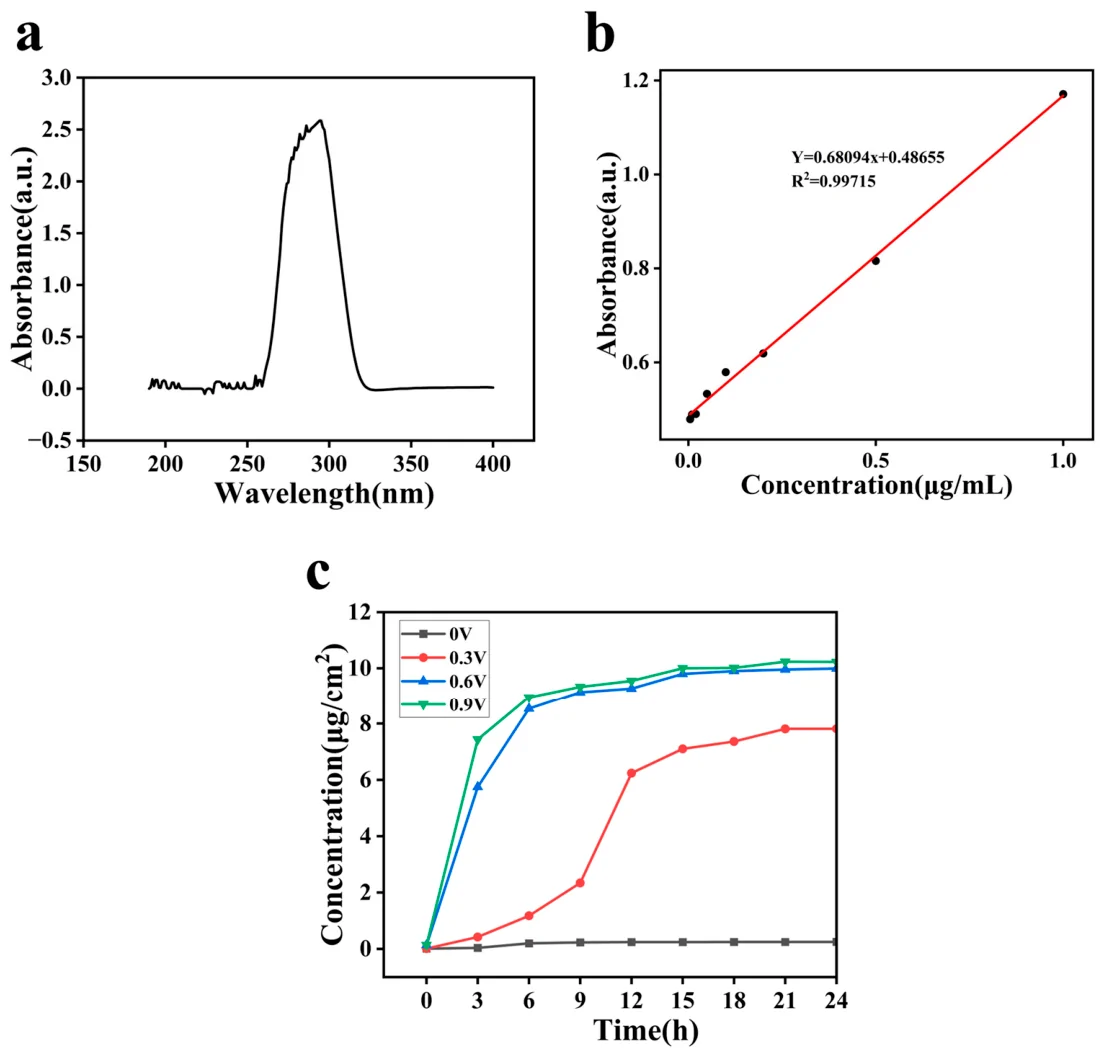
(**a**) UV absorption spectrum of sorafenib; (**b**) calibration curve for sorafenib; (**c**) the electro-release control curve of Sorafenib.

To establish a calibration curve for sorafenib, a stock solution at a concentration of 1000 μg mL^−1^ was first prepared. Its UV–Vis absorption spectrum was recorded over the wavelength range of 200–350 nm using a UV–Vis spectrophotometer. As shown in Figure 3a, sorafenib exhibited a distinct absorption maximum at 285 nm; therefore, this wavelength was selected for subsequent quantitative analysis. A series of sorafenib standard solutions with different concentrations was then prepared, and their absorbance values were measured at 285 nm using a microplate reader. The calibration curve was constructed by plotting absorbance against sorafenib concentration, followed by linear regression analysis. The resulting calibration curve is shown in Figure 3b, and the fitted equation was as follows:

(1) y=0.68094x+0.48655

The correlation coefficient R^2^ = 0.99715 indicates a strong linear relationship between sorafenib concentration and absorbance within the measured concentration range, making it suitable for the quantitative analysis of drug release concentrations in subsequent experiments.

Figure 3c presents the sorafenib release profiles of NiTi−PPy−S under different ES conditions. In the absence of ES (0 V), only limited initial release was observed, followed by a near-plateau, indicating relatively low passive leakage of sorafenib from the coating. At an applied potential of 0.3 V, sorafenib was released relatively rapidly during the first 12 h, after which the release rate gradually decreased. When the applied potential was increased to 0.6 or 0.9 V, a more pronounced initial release occurred within the first 6 h, followed by a slower-release or plateau phase. These findings demonstrated that sorafenib release from NiTi−PPy−S was dependent on the magnitude of the applied ES, with higher potentials producing faster initial release. This potential-dependent behavior may be associated with electrically induced changes in the redox state, charge density, chain conformation, and ion-transport properties of the PPy coating. In conventional PPy−based delivery systems, anionic drug molecules can serve as counterions that compensate for the positive charges generated along the oxidized polymer backbone [34]. Upon ES, redox switching of PPy may alter electrostatic interactions and induce ion exchange, polymer swelling or contraction, and changes in molecular diffusion, thereby facilitating the release of incorporated compounds [35,36]. However, because sorafenib may not function as a conventional anionic dopant under the present release conditions, its release may also involve the diffusion or desorption of drug molecules physically entrapped within the PPy network. Therefore, the observed release behavior likely resulted from the combined effects of PPy redox switching, ion redistribution, structural reorganization of the coating, and enhanced sorafenib diffusion. Collectively, these results confirmed the electrically responsive and potential-dependent release characteristics of NiTi−PPy−S.

To investigate the electrochemical response and drug-loading and release characteristics of PPy coatings, sorafenib-loaded PPy coatings were prepared at different electropolymerization voltages ranging from 0.4 to 0.7 V. Their drug loading efficiency (DLE), cumulative drug release (CDR), and drug loading capacity (DLC) were subsequently evaluated. As shown in Table 2, the electropolymerization voltage significantly affected the sorafenib-loading and release performance of the PPy coatings. As the electropolymerization voltage increased from 0.4 to 0.7 V, the sorafenib loading efficiency gradually increased from 0.53 ± 0.050% to 2.44 ± 0.020%. Meanwhile, the cumulative amount of sorafenib released increased significantly from 1.324 ± 0.122 μg/cm^2^ to 10.522 ± 0.295 μg/cm^2^, while the drug loading capacity increased from 1.487 ± 0.171 μg/cm^2^ to 10.960 ± 0.146 μg/cm^2^. Notably, the PPy coating prepared at 0.7 V exhibited the highest drug loading capacity and cumulative drug release, with a cumulative release amount approximately 7.9 times higher than that of the coating prepared at 0.4 V. Correspondingly, the cumulative release percentage increased from approximately 89.0% at 0.4 V to 96.0% at 0.7 V. These results indicate that the electropolymerization voltage is a key parameter governing the drug loading capacity and electroresponsive drug-release performance of PPy coatings. The enhanced drug loading capacity observed at higher voltages may be attributed to the formation of a more developed PPy network during electropolymerization, thereby creating a larger drug reservoir for subsequent electrically stimulated drug release. The fitted equation is:

(2) $$DLE\;(\%)=\left[\frac{loaded\;drug\;amount}{initial\;drug\;amount}\right]\cdot 100\%$$

### 3.4. Selective Tumor Cell Ablation

**Figure 4:**
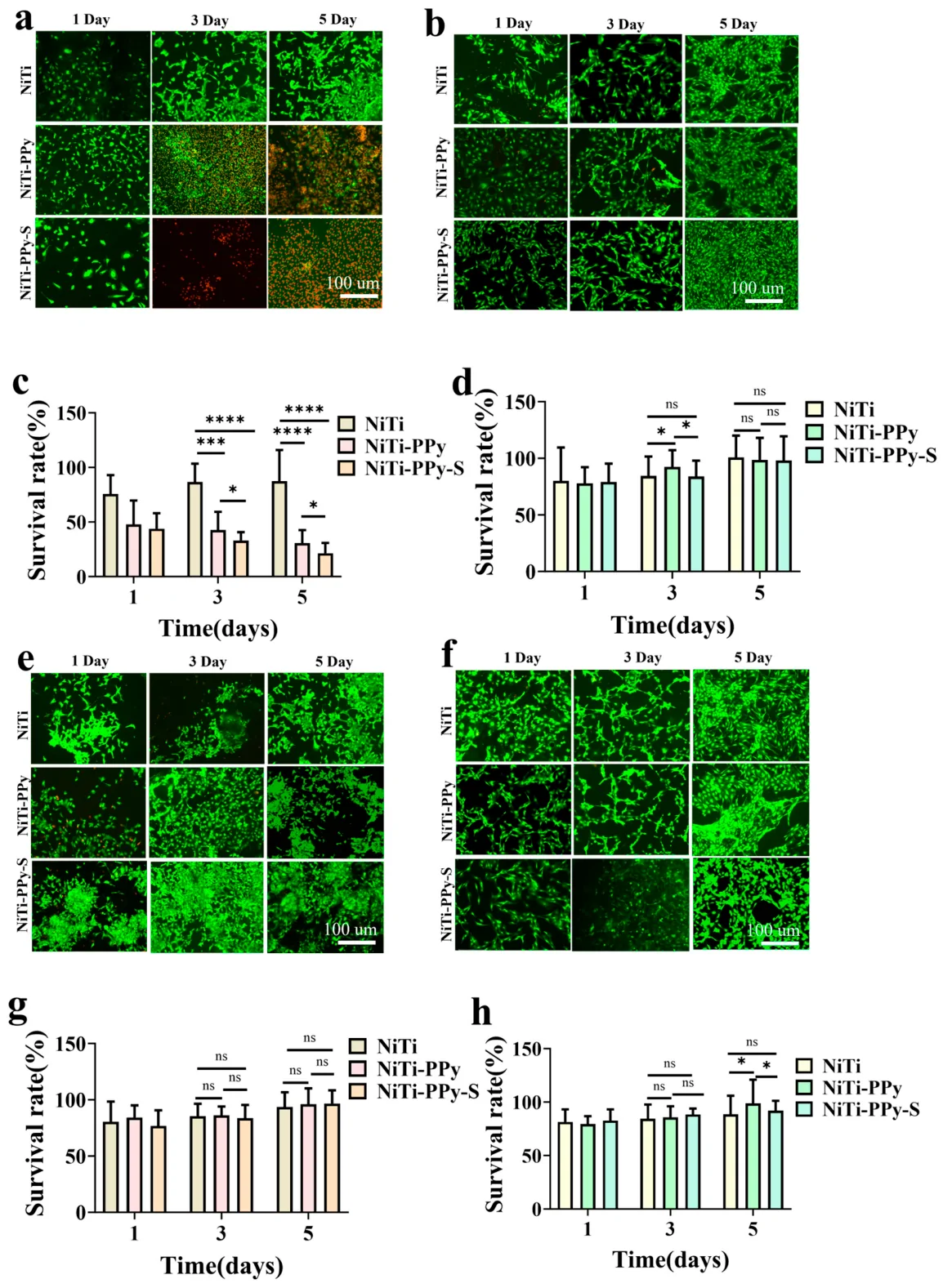
(**a**) Fluorescence images of Calcein-AM/PI staining in Hepa1-6 cells and (**b**) HUVECs cultured on NiTi, NiTi−PPy, and NiTi−PPy−S surfaces for 1, 3, and 5 days under ES conditions; (**c**) corresponding CCK-8 quantitative survival rate analysis for Hepa1-6 cells and (**d**) HUVECs under ES conditions; (**e**) fluorescence images of Calcein-AM/PI staining in (**e**) Hepa1-6 cells and (**f**) HUVECs cultured on NiTi, NiTi−PPy, and NiTi−PPy−S surfaces for 1, 3, and 5 days under non-ES conditions; (**g**) corresponding CCK-8 quantitative survival rate analysis for (**g**) Hepa1-6 cells and (**h**) HUVECs under non-ES conditions. Data are presented as mean ± standard deviation (*n* = 3); * *p* < 0.05, *** *p* < 0.001, **** *p* < 0.0001. Scare bar: 100 μm.

To distinguish ES-mediated cellular effects from the intrinsic cytotoxicity of the material, the cellular responses to NiTi, NiTi−PPy, and NiTi−PPy−S were evaluated in the presence (+ES) and absence (−ES) of ES. Under +ES conditions, live/dead staining revealed a time-dependent increase in Hepa1-6 cell death over the culture period (Figure 4a,b). The corresponding CCK-8 results for Hepa1-6 cells and HUVECs are shown in Figure 4c,d, respectively. After 5 days of culture, the relative viability of Hepa1-6 cells in the relevant treatment group decreased to approximately 20%, indicating a pronounced ES-mediated antitumor effect. In contrast, under −ES conditions, both Hepa1-6 cells and HUVECs exhibited favorable growth and survival on the surfaces of NiTi, NiTi−PPy, and NiTi−PPy−S (Figure 4e,f). Predominantly green fluorescence with only sparse red fluorescence was observed in all groups, indicating a high proportion of viable cells and minimal cell death. Consistent with the live/dead staining results, the CCK-8 assay showed that the relative metabolic activity of both cell types remained at or above 89% under −ES conditions (Figure 4g,h). These findings suggested that the materials exhibited favorable baseline cytocompatibility in the absence of ES, whereas ES induced a marked antitumor response in Hepa1-6 cells.

**Figure 5:**
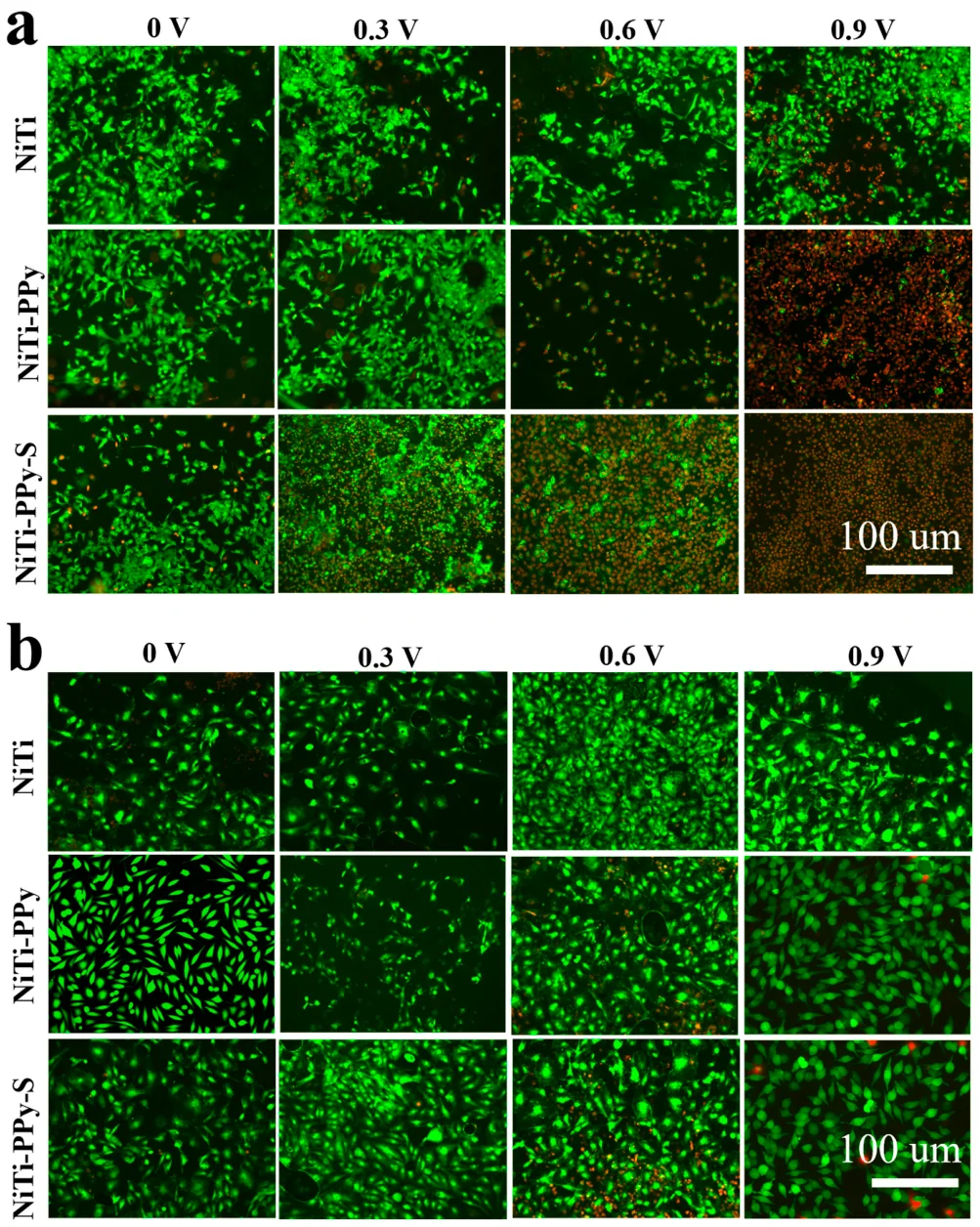
Fluorescent images of live/dead cell staining in (**a**) Hepa1-6 cells and (**b**) HUVECs 24 h after treatment with different voltages (0 V, 0.3 V, 0.6 V, 0.9 V). Scare bar: 100 μm.

Under ES, Hepa1-6 cells and HUVECs exhibited distinct cellular responses on the different sample surfaces. As shown in Figure 5a, the viability of Hepa1-6 cells progressively decreased as the applied electrical stimulus increased from 0 to 0.9 V, indicating an ES-dependent cytotoxic response. At 0.6 and 0.9 V, red fluorescence predominated, accompanied by a marked reduction in green fluorescence, suggesting extensive Hepa1-6 cell death. In contrast, HUVECs retained relatively high viability across the different material surfaces over the same range of applied ES (Figure 5b). This differential response suggested that the electroresponsive coating exerted a stronger cytotoxic effect on Hepa1-6 cells than on HUVECs under ES conditions. Increasing the applied ES may enhance interfacial electrochemical reactions, charge transfer, and electrically triggered sorafenib release, thereby increasing tumor cell damage. Meanwhile, HUVECs appeared to exhibit greater tolerance to the applied electrical conditions, although the mechanisms underlying this differential sensitivity require further investigation. Collectively, these findings indicated that NiTi−PPy−S could function as an electrically activated therapeutic interface. In the absence of ES, the coating exhibited favorable cytocompatibility, whereas under ES conditions, it induced pronounced Hepa1-6 cell death while largely preserving HUVECs’ viability.

**Figure 6:**
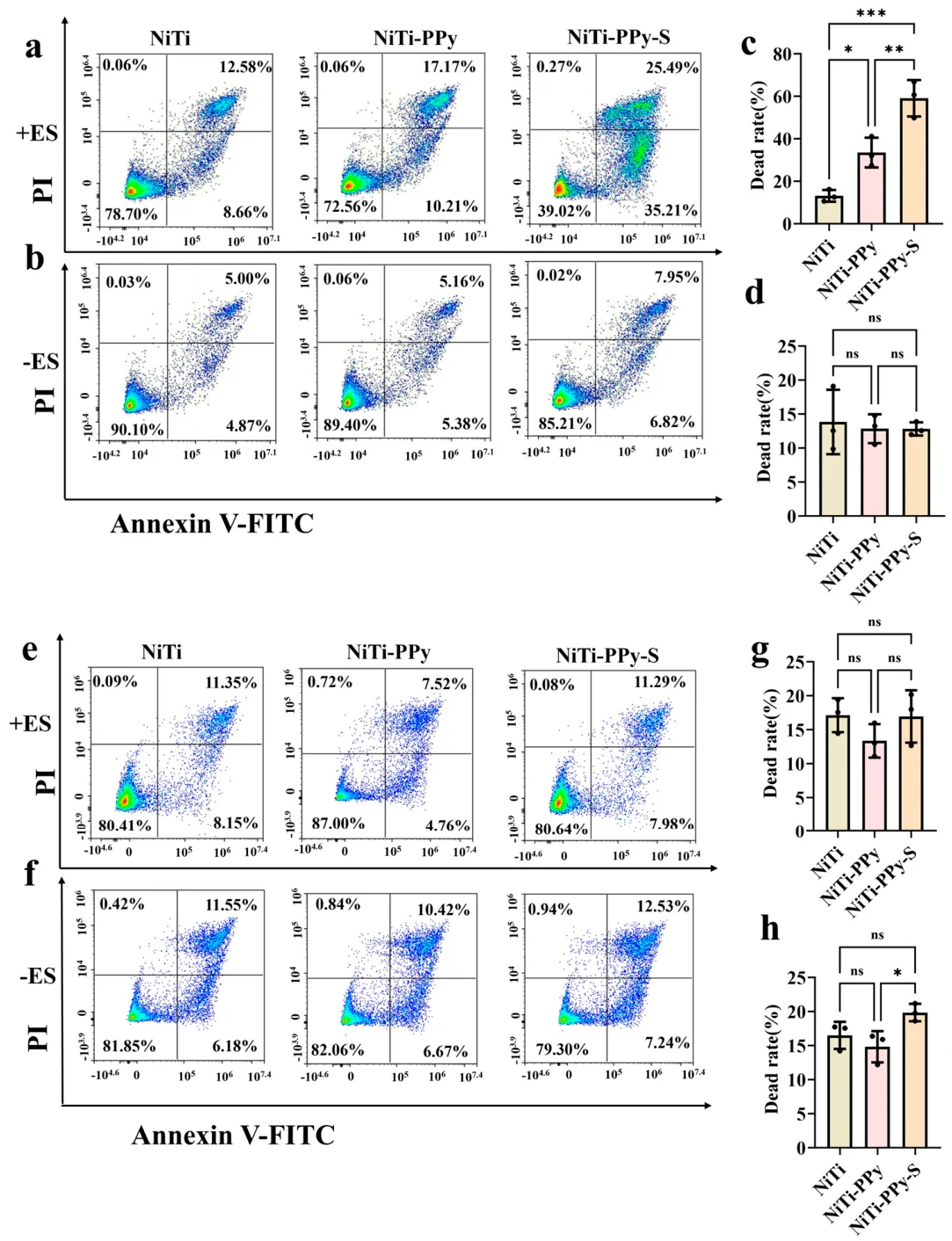
(**a**) Annexin V-FITC/PI flow cytometry plots of Hepa1-6 cells treated with ES in the NiTi, NiTi−PPy, and NiTi−PPy−S groups, and (**b**) those treated without ES. (**c**) Quantitative analysis of total cell death in Hepa1-6 cells treated with ES and (**d**) those treated without ES. (**e**) Annexin V-FITC/PI flow cytometry plots of HUVECs treated with ES in the NiTi, NiTi−PPy, and NiTi−PPy−S groups, and (**f**) those treated without ES. (**g**) Quantitative analysis of total cell death in HUVECs treated with ES and (**h**) those treated without ES. Data are presented as mean ± standard deviation (*n* = 3); * *p* < 0.05, ** *p* < 0.01, *** *p* < 0.001.

Flow cytometric analysis further demonstrated that NiTi−PPy−S induced a pronounced pro-apoptotic response in Hepa1-6 cells under ES. In the +ES−NiTi−PPy−S group, the proportion of viable Hepa1-6 cells decreased to approximately 39%, accompanied by marked increases in the proportions of early apoptotic and late apoptotic/secondary necrotic cells (Figure 6a,c). Accordingly, the combined proportion of nonviable and apoptotic cell populations reached approximately 61%, indicating a strong apoptosis-inducing effect under ES conditions. As shown in Figure 6b,d, in the absence of ES, the proportion of viable Hepa1-6 cells remained at approximately 85–90%, and the combined proportion of apoptotic and necrotic cells in the NiTi−PPy−S group was only approximately 12.77%. These results indicated that NiTi−PPy−S exhibited limited intrinsic cytotoxicity without electrical activation. For HUVECs, the majority of cells remained within the viable-cell quadrant, regardless of whether ES was applied (Figure 6e,f). Quantitative analysis showed that the combined proportion of apoptotic and necrotic HUVECs was approximately 20%, with no statistically significant differences among the treatment groups (Figure 6g,h). Collectively, these findings suggested that the pronounced cytotoxic response observed in Hepa1-6 cells was not primarily attributable to the intrinsic toxicity of the coating but was predominantly associated with ES activation of the NiTi−PPy−S therapeutic interface.

**Figure 7:**
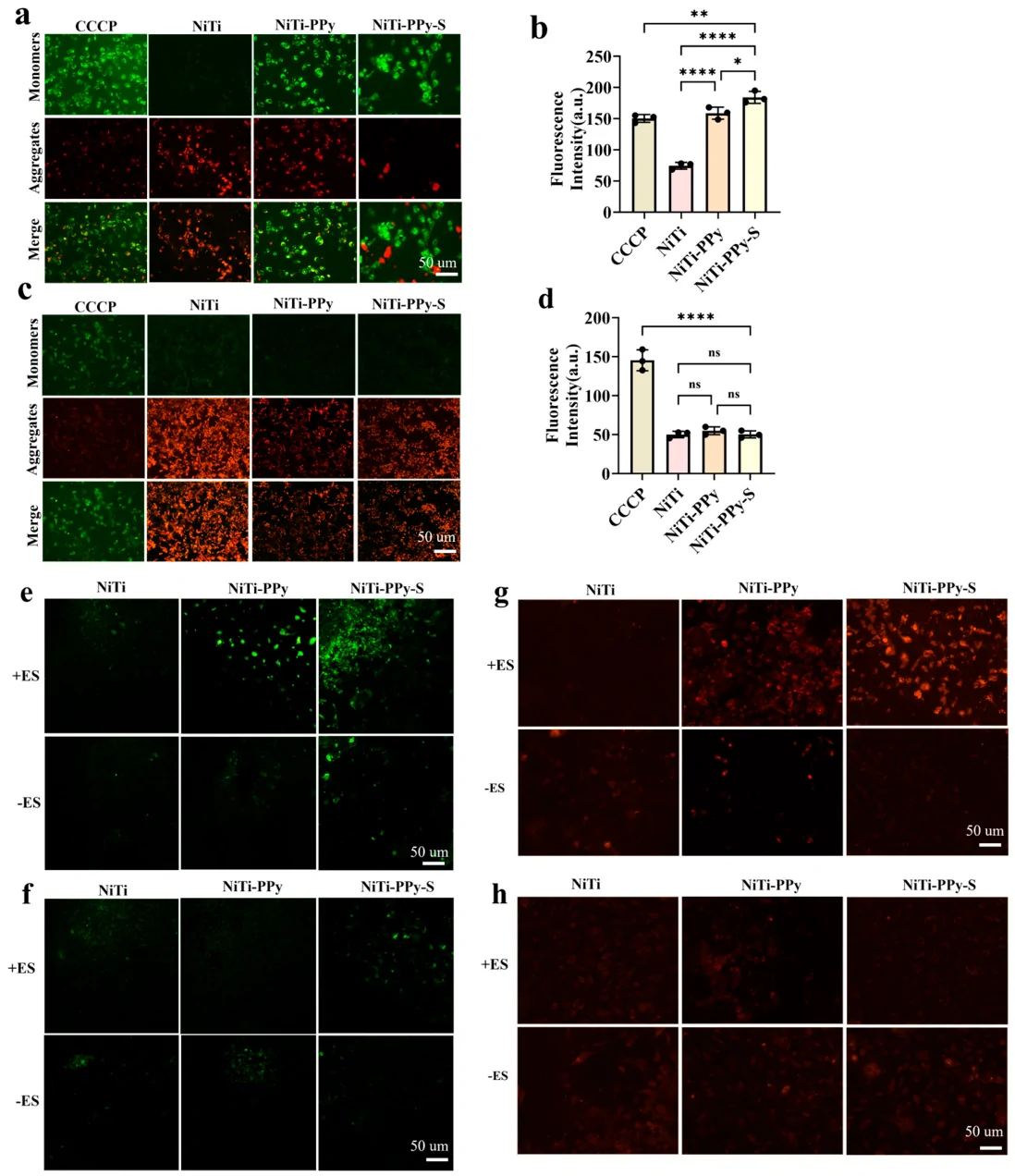
(**a**) Representative microscopic images of Hepa1-6 cells and (**c**) HUVECs stained with the JC-1 fluorescent probe; (**b**,**d**) show quantitative analyses of the average fluorescence intensity of green fluorescence in the two cell types, respectively. (**e**) Calcium influx in Hepa1-6 cells. (**f**) Calcium influx in HUVECs. (**g**) Mitochondrial calcium influx in Hepa1-6 cells. (**h**) Mitochondrial calcium influx in HUVECs. Data are presented as mean ± standard deviation (*n* = 3); * *p* < 0.05, ** *p* < 0.01, **** *p* < 0.0001.

To investigate the mechanism underlying the apoptosis of tumor cells, mitochondrial membrane potential (Δψm) was assessed using JC-1 staining. JC-1 fluorescence imaging and quantitative analysis showed that, under ES, the NiTi−PPy−S system induced a more pronounced loss of Δψm in Hepa1-6 cells than in HUVECs. As shown in Figure 7a, Hepa1-6 cells in the +ES−NiTi−PPy−S group exhibited predominantly green fluorescence derived from JC-1 monomers, accompanied by a marked reduction in red fluorescence from JC-1 aggregates. Quantitative analysis revealed a significantly reduced red-to-green fluorescence intensity ratio compared with the NiTi group (Figure 7b), indicating substantial mitochondrial membrane depolarization in Hepa1-6 cells. Because loss of Δψm is an early event associated with mitochondrial dysfunction and apoptosis, these findings suggested that NiTi−PPy−S under ES initiated mitochondria-associated apoptotic signaling in Hepa1-6 cells. In contrast, HUVECs retained predominantly red JC-1 aggregate fluorescence across the experimental groups (Figure 7c), indicating relatively preserved mitochondrial membrane potential. Quantitative analysis further showed that the red-to-green fluorescence ratio remained comparatively high, with only limited changes among the treatment groups (Figure 7d). These results suggested that the ES-activated NiTi−PPy−S system caused greater mitochondrial dysfunction in Hepa1-6 cells than in HUVECs under the tested conditions.

The effects of ES on intracellular Ca^2+^ levels in Hepa1-6 cells and HUVECs cultured on NiTi, NiTi−PPy, and NiTi−PPy−S surfaces were evaluated using the fluorescent Ca^2+^ indicator Fluo-4 AM. As shown in Figure 7e, the three materials induced distinct changes in intracellular Ca^2+^-associated fluorescence in Hepa1-6 cells under ES conditions. In the bare NiTi group, the fluorescence signal remained weak and showed little change compared with the corresponding −ES group, suggesting that the native NiTi surface had a limited capacity to mediate ES-induced intracellular Ca^2+^ elevation. In contrast, Hepa1-6 cells cultured on NiTi−PPy exhibited markedly enhanced green fluorescence after ES, indicating that the conductive PPy coating facilitated the transmission of electrical stimuli across the material-cell interface and promoted an increase in intracellular Ca^2+^ levels. A similarly enhanced fluorescence response was observed in the NiTi−PPy−S group, with widespread regions of intense green fluorescence. These findings suggested that PPy modification improved the interfacial electroactivity of NiTi and enhanced ES-induced Ca^2+^ signaling in Hepa1-6 cells. As shown in Figure 7f, HUVECs exhibited relatively weak green fluorescence in the NiTi, NiTi−PPy, and NiTi−PPy−S groups under the same +ES conditions. No statistically significant differences were observed compared with the corresponding control or −ES groups, suggesting that ES induced only limited changes in intracellular Ca^2+^ levels in HUVECs under the tested conditions. Rhod-2 AM staining revealed a comparable pattern of mitochondrial Ca^2+^-associated fluorescence. As shown in Figure 7g, ES-treated Hepa1-6 cells cultured on PPy-modified surfaces exhibited enhanced Rhod-2 fluorescence, indicating increased mitochondrial Ca^2+^ accumulation. Excessive mitochondrial Ca^2+^ accumulation can disrupt mitochondrial homeostasis, promote mitochondrial membrane depolarization, and activate downstream apoptotic signaling. These results therefore suggested that the ES-induced elevation of intracellular Ca^2+^ in Hepa1-6 cells was accompanied by increased mitochondrial Ca^2+^ uptake, potentially contributing to mitochondrial dysfunction and apoptosis. In contrast, HUVECs exhibited only weak Rhod-2 fluorescence across the NiTi, NiTi−PPy, and NiTi−PPy−S groups under identical +ES conditions, with no statistically significant differences relative to the corresponding control or −ES groups (Figure 7h). Collectively, these findings indicated that the ES-activated PPy-based coatings induced more pronounced intracellular and mitochondrial Ca^2+^ responses in Hepa1-6 cells than in HUVECs.

**Figure 8:**
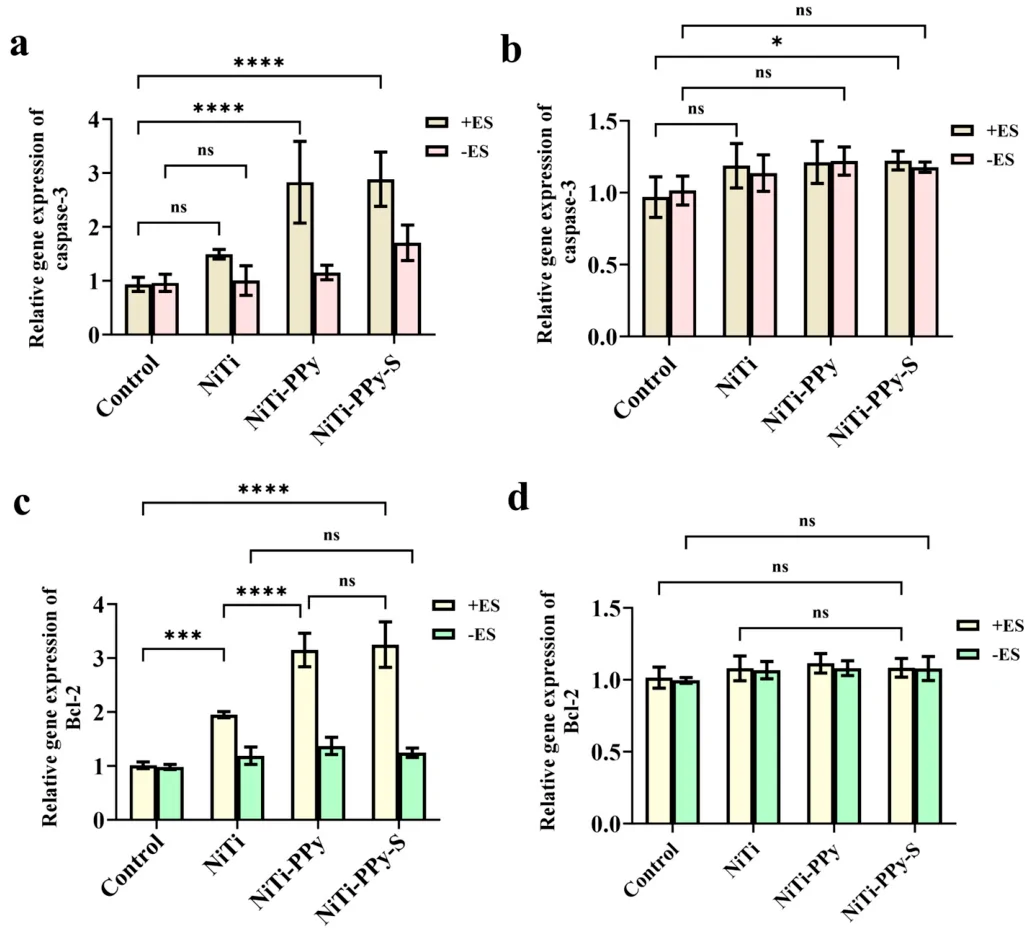
(**a**) The effects of various components on Caspase-3 gene expression and (**c**) Bcl-2 gene expression in Hepa1-6 cells under conditions ±ES. (**b**) The effects of various components on Caspase-3 gene expression and (**d**) Bcl-2 gene expression in HUVECs under conditions ±ES. Data are presented as mean ± standard deviation (*n* = 3); * *p* < 0.05, *** *p* < 0.001, **** *p* < 0.0001.

RT-qPCR analysis revealed changes in the transcription of apoptosis-related genes, including the pro-apoptotic gene Caspase3 and the anti-apoptotic gene Bcl-2. Compared with the NiTi group, the NiTi−PPy and NiTi−PPy−S groups exhibited markedly increased Caspase3 mRNA expression under ES. As shown in Figure 8a,b, Hepa1-6 cells cultured on the NiTi−PPy−S surface exhibited the greatest increase in Caspase3 expression following ES treatment, reaching approximately 3.1-fold relative to the control group. However, Bcl-2 expression also increased to approximately 3.5-fold, suggesting that the ES-activated NiTi−PPy−S interface induced a complex transcriptional response involving both pro-apoptotic signaling and a potential compensatory anti-apoptotic response. In the absence of ES, the expression levels of both genes remained relatively low across the different material groups. In contrast, the expression levels of Caspase-3 and Bcl-2 in HUVECs remained relatively stable in the presence and absence of ES, with no statistically significant differences among the treatment groups (Figure 8c,d). Collectively, these findings indicated that ES-activated NiTi−PPy−S induced a pronounced upregulation of Caspase-3 transcription in Hepa1-6 cells, whereas apoptosis-related gene expression in HUVECs was largely preserved.

**Figure 9:**
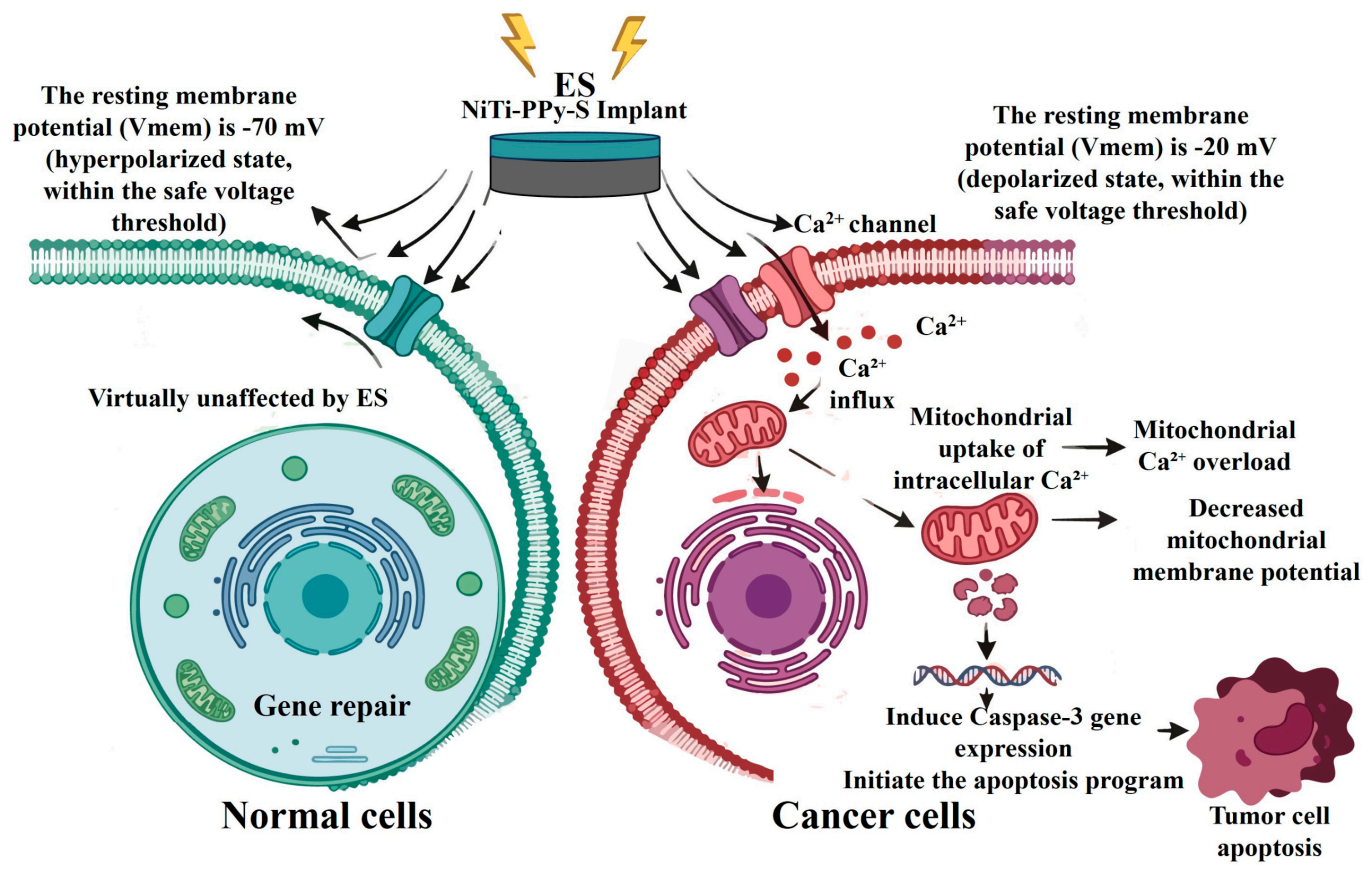
Schematic diagram of the mechanism by which NiTi−PPy−S electro-responsive coatings induce apoptosis in tumor cells.

Previous studies have reported differences in resting membrane potential (Vmem) between nonmalignant and malignant cells [37]. Figure 9 illustrates the hypothesized bioelectrical mechanism underlying the preferential apoptotic response of tumor cells to the NiTi−PPy−S electroresponsive coating. Nonmalignant cells generally maintain a more negative resting membrane potential, often near −70 mV, and therefore exist in a relatively hyperpolarized state. In contrast, many tumor cells exhibit a less negative resting membrane potential, frequently approaching −20 mV, and are consequently more depolarized. This depolarized state may render tumor cells more sensitive to externally applied electrical stimuli and associated disturbances in ion homeostasis. Upon ES, tumor cells may experience greater changes in transmembrane potential and Ca^2+^ signaling, thereby increasing their susceptibility to mitochondrial dysfunction and apoptosis. By comparison, nonmalignant cells may exhibit greater tolerance to the applied electrical conditions, potentially owing to differences in membrane properties, ion-channel activity, and cellular stress-response capacity. Studies involving pulsed electric-field exposure have also suggested that nonmalignant cells may display greater resistance to membrane permeabilization and more efficient recovery of membrane integrity than certain tumor cells [38]. Frandsen et al. compared intracellular Ca^2+^ responses in normal fibroblasts and several tumor-cell types following electrical pulse treatment [39]. Their findings suggested that cell-type-dependent electrophysiological properties can influence susceptibility to electric-field-induced membrane permeabilization and Ca^2+^ entry. Accordingly, the stronger intracellular and mitochondrial Ca^2+^ responses observed in Hepa1-6 cells may be partly related to their bioelectrical state, whereas HUVECs appeared to be comparatively resistant under the ES conditions used in this study.

Based on the proposed bioelectrical mechanism, the NiTi−PPy−S electroresponsive coating enhances the transmission of ES at the material-cell interface, thereby promoting an elevation in intracellular Ca^2+^ channels in tumor cells. The increased intracellular Ca^2+^ is accompanied by enhanced mitochondrial Ca^2+^ uptake, resulting in mitochondrial Ca^2+^ accumulation, disruption of mitochondrial homeostasis, and mitochondrial membrane depolarization (Δψm loss). These mitochondrial alterations are associated with the activation of mitochondria-mediated apoptotic signaling, accompanied by upregulation of Caspase-3 expression, ultimately leading to apoptotic death of tumor cells.

Pan et al. developed a flexible microneedle-array-integrated interdigital electrode capable of generating an alternating electric field within tumor tissue. In B16F10 melanoma cells, ES markedly increased cytosolic Ca^2+^ levels and subsequently induced mitochondrial Ca^2+^ overload and loss of mitochondrial membrane potential. These events ultimately induced necrotic-like cell death with hallmarks of immunogenic cell death, including extracellular ATP and HMGB1 release and cell-surface calreticulin exposure [40,41]. Meanwhile, other studies involving calcium electroporation and pulsed electric fields have further demonstrated that mitochondrial damage caused by Ca^2+^ overload can be converted into apoptotic signaling under specific tumor-cell contexts and stimulation parameters. For example, calcium electroporation markedly activated caspase-3/7 in rhabdomyosarcoma cells, with apoptosis identified as the predominant mode of cell death [42]. In DU145 prostate cancer cells, calcium electroporation similarly enhanced caspase-3 activation and promoted apoptosis [43]. In addition, nanosecond pulsed electric fields have been shown to increase intracellular Ca^2+^ levels, induce loss of mitochondrial membrane potential and oxidative stress, and activate mitochondria-associated apoptotic pathways in hepatocellular carcinoma and lung cancer cells [44]. Therefore, in the NiTi−PPy−S system, sustained Ca^2+^ influx may induce mitochondrial Ca^2+^ overload and collapse of the mitochondrial membrane potential, subsequently activating mitochondrial apoptotic signaling and the proteolytic cleavage of caspase-3, thereby promoting apoptosis in tumor cells.

### 3.5. In Vivo Antitumor Activity

**Figure 10:**
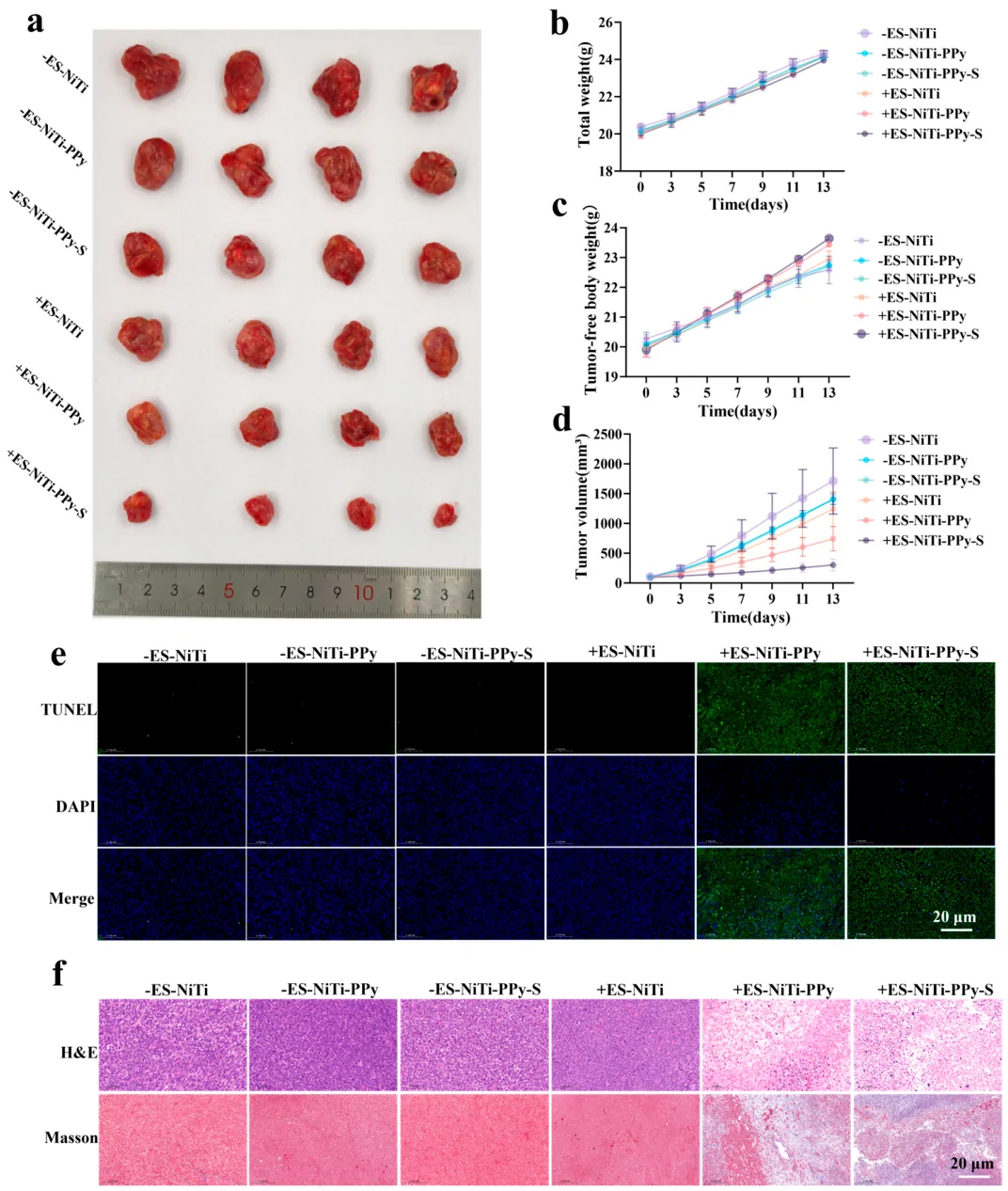
(**a**) Photographs of ex vivo tumor morphology; (**b**) Curve showing changes in total body weight of mice (*n* = 6); (**c**) Curve showing tumor volume growth (*n* = 6); (**d**) Curve showing changes in tumor-free body weight of mice (*n* = 6); (**e**) Histopathological analysis of tumor tissues from different treatment groups; (**f**) Analysis of immunofluorescence staining of tumor tissue following ES ablation. Data are presented as the mean ± standard deviation.

The in vivo antitumor efficacy of NiTi−PPy−S was further evaluated in a Hepa1-6 tumor-bearing mouse model. Following implantation of the different samples, ES was applied to the designated treatment groups. Representative photographs of excised tumors are shown in Figure 10a. Mice in the −ES groups exhibited larger tumors, indicating continued tumor progression during the experimental period. In contrast, tumor growth was markedly suppressed in the ES-treated groups, with the greatest inhibition observed in the +ES−NiTi−PPy−S group. Quantitative analysis of tumor growth curves further demonstrated that the +ES−NiTi−PPy−S group exhibited the slowest increase in tumor volume throughout the treatment period, indicating an enhanced therapeutic effect resulting from the combination of the electroresponsive sorafenib-loaded PPy coating and ES. Systemic tolerability was evaluated by monitoring body weight throughout the treatment period. The total body weight of mice increased gradually in all groups, from approximately 20 g to 24 g. Because total body weight included the contribution of tumor mass, tumor-free body weight was further analyzed. As shown in Figure 10b–d, tumor-free body weight also increased steadily throughout the study, suggesting that the treatments did not cause obvious treatment-related body weight loss and were generally well tolerated under the experimental conditions.

Histological analyses further supported the in vivo antitumor efficacy of the ES-activated NiTi−PPy−S system. As shown in Figure 10e, TUNEL fluorescence staining revealed a marked increase in TUNEL-positive signals in the +ES−NiTi−PPy−S group, indicating extensive DNA fragmentation associated with apoptosis. Masson’s trichrome staining further showed marked disruption of the tumor extracellular matrix and loss of tissue integrity following ES treatment, with the most pronounced histological alterations observed in the +ES−NiTi−PPy−S group. Together with the tumor-growth data, these findings supported the ability of the NiTi−PPy−S system combined with ES to induce extensive tumor-cell injury and apoptosis in vivo. H&E staining further revealed distinct histopathological differences among the treatment groups (Figure 10f). In the −ES control groups, tumor cells remained densely arranged, with relatively intact nuclei and preserved tissue architecture, consistent with the presence of largely viable tumor tissue. In the +ES−NiTi group, only limited histological damage was observed, suggesting that ES applied through bare NiTi produced a relatively weak antitumor response under the tested conditions. In contrast, tumors in the +ES−NiTi−PPy and +ES−NiTi−PPy−S groups exhibited extensive pale eosinophilic regions, nuclear pyknosis and fragmentation, reduced cellular density, and pronounced disruption of tissue architecture, indicating substantial treatment-induced tumor injury. These histopathological alterations were most extensive in the +ES−NiTi−PPy−S group, consistent with its superior inhibition of tumor growth.

**Figure 11:**
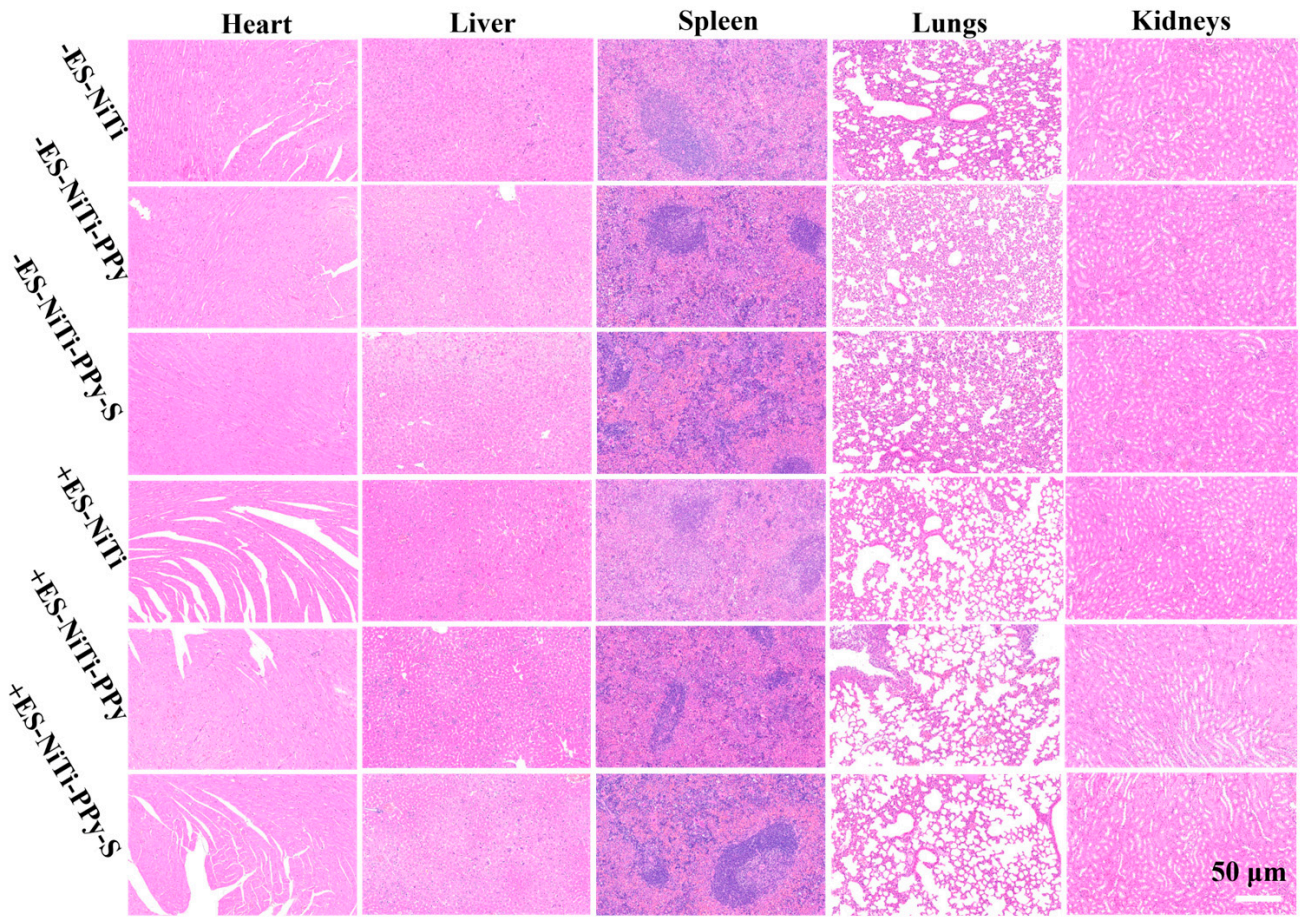
Histopathological analysis of tumor tissues from different treatment groups.

The in vivo systemic tolerability of the therapeutic system was evaluated by histological examination of major organs, including the heart, liver, spleen, lungs, and kidneys. H&E-stained tissue sections were examined using light microscopy. As shown in Figure 11, compared with the corresponding −ES control groups, mice receiving the different implants in combination with ES exhibited no apparent histopathological abnormalities in the examined organs. The overall tissue architecture remained intact, with no evident inflammatory cell infiltration, structural disruption, or treatment-related lesions. These findings indicated that the NiTi, NiTi−PPy, and NiTi−PPy−S implants, with or without ES, did not induce detectable major-organ toxicity under the experimental conditions, supporting their favorable preliminary in vivo biocompatibility and systemic tolerability.

## 4. Conclusions

In this study, we developed a sorafenib-loaded, PPy coating on a NiTi alloy substrate. Physicochemical and electrochemical characterization demonstrated that PPy modification enhanced the interfacial electroactivity of the NiTi substrate. After sorafenib loading, the resulting NiTi−PPy−S platform maintained favorable electrochemical properties and enabled electrically triggered drug release. In vitro, NiTi−PPy−S exhibited favorable baseline cytocompatibility in the absence of ES. Upon ES, Hepa1-6 cells cultured on NiTi−PPy−S showed a significant reduction in viability, accompanied by increased intracellular and mitochondrial Ca^2+^ levels, mitochondrial membrane depolarization, and upregulation of Caspase-3 expression. By comparison, HUVECs exhibited substantially weaker Ca^2+^ and mitochondrial responses under the same treatment conditions, suggesting a preferential antitumor effect of the ES-activated NiTi−PPy−S platform. In vivo, NiTi−PPy−S combined with ES markedly inhibited subcutaneous tumor growth and increased tumor-associated DNA fragmentation and histopathological damage compared with the other treatment groups. Moreover, no obvious treatment-related body-weight loss or major-organ histopathological abnormalities were detected during the experimental period, supporting the preliminary systemic tolerability of this therapeutic strategy.

Electroresponsive biomedical platforms have been widely investigated for electrically triggered drug delivery, enabling on-demand and electrically regulated release of therapeutic agents [34,45]. Separately, pulsed electric-field technologies, such as irreversible electroporation, have been developed for nonthermal tumor ablation [46]. PPy-based coatings can release encapsulated drugs in response to an applied voltage, whereas electrically active microneedle devices mainly use local electric fields to induce tumor cell damage. The NiTi−PPy−S system integrates these two functions into a single implant. The NiTi substrate provides the mechanical properties required for interventional applications, while the PPy coating enables electrically induced sorafenib release and conducts the applied ES to the surrounding tumor tissue. This design therefore combines electrically controlled local drug delivery with ES-mediated tumor cell killing and may be particularly suitable for malignant lesions located within or adjacent to blood vessels.

Collectively, these findings establish a multifunctional therapeutic platform that integrates a NiTi interventional implant, an electrically responsive PPy coating, localized sorafenib delivery, and ES-mediated tumor ablation. The results provide preliminary proof-of-concept evidence for the local antitumor potential of this strategy. Nevertheless, because the in vivo efficacy was evaluated exclusively in a subcutaneous tumor model, its translational relevance to PVTT remains to be determined. Further studies using orthotopic hepatocellular carcinoma models and PVTT-specific models are therefore required to validate its therapeutic efficacy, safety, and clinical applicability in PVTT and other malignant vascular obstructive lesions. Furthermore, although the PPy coating exhibited satisfactory structural integrity and electrochemical performance under the conditions examined in this study, its long-term durability remains to be established. Future engineering studies should systematically evaluate coating adhesion, polymer degradation, electrochemical stability, and the reproducibility of drug release under repeated ES and prolonged exposure to physiologically relevant environments.

## Figures and Tables

**Table 1:** Abbreviations for different sample components.

Sample Code	Surface Composition	Sorafenib Loading Dose	ES
NiTi	Polished and etched NiTi alloy	None	±ES
NiTi−PPy	Deposition of a PPy coating on a NiTi	None	±ES
NiTi−PPy−S	NiTi-coated PPy coatings loaded with sorafenib	Yes	±ES

**Table 2:** DLE, CDR, DLC and Cumulative release of sorafenib-loaded PPy coatings prepared at different electropolymerization voltages.

Electropolymerization Voltage (V)	DLE (%)	CDR (μg/cm^2^)	DLC (μg/cm^2^)	Cumulative Release (%)
0.4	0.53 ± 0.050	1.324 ± 0.122	1.487 ± 0.171	89% ± 0.030
0.5	0.61 ± 0.030	1.691 ± 0.180	1.858 ± 0.120	91% ± 0.010
0.6	1.96 ± 0.060	8.177 ± 0.138	8.698 ± 0.189	94% ± 0.030
0.7	2.44 ± 0.020	10.522 ± 0.295	10.960 ± 0.146	96% ± 0.02

## Data Availability

The original contributions presented in this study are included in the article. Further inquiries can be directed to the corresponding authors.

## Abbreviations

The following abbreviations are used in this manuscript:

+ES	Apply an electrical stimulus
−ES	No electrical stimulation is applied
NiTi	Nickel–titanium
PPy	Polypyrrole
S	Sorafenib
Py	Pyrrole monomer
DAMPs	Damage-associated molecular patterns
